# Application of Precision Agriculture Technologies for Sustainable Crop Production and Environmental Sustainability: A Systematic Review

**DOI:** 10.1155/2024/2126734

**Published:** 2024-10-09

**Authors:** Sewnet Getahun, Habtamu Kefale, Yohannes Gelaye

**Affiliations:** ^1^Department of Plant Science, College of Agriculture and Natural Resource, Debre Markos University, P.O. Box: 269, Debre Markos, Amhara, Ethiopia; ^2^Department of Horticulture, College of Agriculture and Natural Resource, Debre Markos University, P.O. Box: 269, Debre Markos, Amhara, Ethiopia

**Keywords:** environmental sustainability, precision agriculture technologies, precision farming, sustainable crop production

## Abstract

Precision agriculture technologies (PATs) transform crop production by enabling more sustainable and efficient agricultural practices. These technologies utilize data-driven approaches to optimize the management of crops, soil, and resources, thus enhancing both productivity and environmental sustainability. This article reviewed the application of PATs for sustainable crop production and environmental sustainability around the globe. Key components of PAT include remote sensing, GPS-guided equipment, variable rate technology (VRT), and Internet of Things (IoT) devices. Remote sensing and drones deliver high-resolution imagery and data, enabling precise monitoring of crop health, soil conditions, and pest activity. GPS-guided machinery ensures accurate planting, fertilizing, and harvesting, which reduces waste and enhances efficiency. VRT optimizes resource use by allowing farmers to apply inputs such as water, fertilizers, and pesticides at varying rates across a field based on real-time data and specific crop requirements. This reduces over-application and minimizes environmental impact, such as nutrient runoff and greenhouse gas emissions. IoT devices and sensors provide continuous monitoring of environmental conditions and crop status, enabling timely and informed decision-making. The application of PAT contributes significantly to environmental sustainability by promoting practices that conserve water, reduce chemical usage, and enhance soil health. By enhancing the precision of agricultural operations, these technologies reduce the environmental impact of farming, while simultaneously boosting crop yields and profitability. As the global demand for food increases, precision agriculture offers a promising pathway to achieving sustainable crop production and ensuring long-term environmental health.

## 1. Introduction

Precision agriculture technologies (PATs) are revolutionizing the agricultural sector by enhancing productivity, efficiency, and sustainability [1]. These technologies leverage advanced tools such as GPS, Internet of Things (IoT), drones, and data analytics to monitor and manage crop production with unprecedented accuracy [2]. By optimizing inputs such as water, fertilizers, and pesticides, precision agriculture minimizes waste, reduces environmental impact, and promotes sustainable farming practices [3]. This approach not only increases crop yields but also contributes to the long-term health of the environment by preserving natural resources and reducing pollution.

Globally, the adoption of PATs is accelerating as they help address challenges such as climate change, resource scarcity, and food security [4]. In developed countries, significant investments in research and infrastructure have facilitated widespread implementation of these technologies, leading to more resilient and sustainable agricultural systems [5]. Emerging economies are also beginning to adopt precision agriculture, albeit at a slower pace, due to the high initial costs and the need for technical expertise [6]. In developing countries, precision agriculture holds great promise for transforming the continent's agricultural landscape [7]. Adoption is growing, driven by initiatives from international organizations and local governments that aim to integrate advanced technologies such as GPS, IoT, and drones into agriculture [8]. The Ethiopian government, with various stakeholders, is working to modernize agriculture by promoting the use of advanced technologies [9]. Projects focused on improving data collection, soil health management, and water use efficiency are being implemented to boost crop yields and ensure environmental sustainability [10]. Despite these efforts, the full potential of precision agriculture in Ethiopia remains untapped, necessitating further investment and capacity building to overcome existing barriers [11]. Limited technology, poor infrastructure, and lack of technical knowledge hinder precision agriculture in Africa, but advancing digital farming offers promising future prospects [12].

Recent advancements in cloud computing, IoT, and technologies like robotics and big data drive the fourth agricultural revolution, Agriculture 4.0 [13]. Agriculture 4.0 leverages these technologies to create a smarter, more efficient, and environmentally responsible sector, focusing on economic, social, and environmental sustainability [14]. By applying modern technologies for real-time data processing, analysis, and decision-making, Agriculture 4.0 optimizes production, supply chain, and logistics performance in an ethical manner [15]. Regarding social benefits, Agriculture 4.0 aims to make agrifood systems more sustainable by reducing food losses and waste and improving food security to end hunger and malnutrition worldwide [16]. Thus, this work aimed to review the application of PATs for sustainable crop production and environmental sustainability.

## 2. Review Methodology

**Figure 1:**
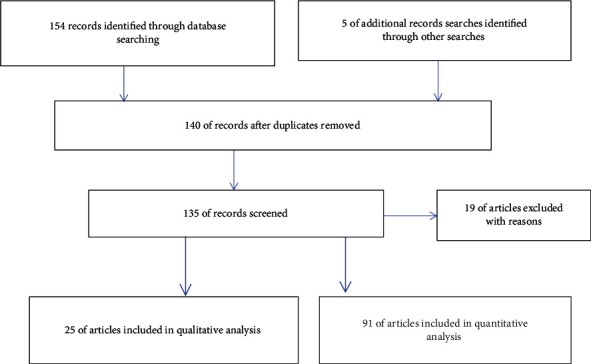
PRISMA flow diagram.

In this systematic review, we explored the application of PATs for sustainable crop production and environmental sustainability by conducting a comprehensive search across major databases including Web of Science, Scopus, and Google Scholar (Figure 1). We utilized index terms such as “PATs,” “sustainable crop production,” “environmental sustainability,” and “precision farming,” focusing on peer-reviewed articles published in English since 2015. We included materials that directly addressed the impact of these technologies on crop production and sustainability, incorporating original research, systematic reviews, and meta-analyses from diverse geographic locations to ensure a global perspective. The exclusion criteria involved materials unrelated to the specified technologies, those in languages other than English, research with insufficient empirical data, and studies from regions with significantly different agricultural practices or climatic conditions. In addition, we removed duplicate records and publications with overlapping content to maintain the review integrity. We performed a synthesis of the selected studies, assessing qualities using different programs.

## 3. Overview of Application of PATs

PATs have revolutionized farming practices worldwide by integrating advanced technologies such as GPS, sensors, drones, and data analytics [17]. These technologies enable farmers to optimize field-level management concerning crop farming. The global adoption of precision agriculture is driven by the need to increase productivity, reduce environmental impact, and enhance sustainability [18]. Countries such as the United States, Canada, Australia, and several European nations have been at the forefront, leveraging innovations such as variable rate technology (VRT), remote sensing, and automated machinery [19]. These advancements allow for real-time monitoring and management of crops, leading to higher yields and more efficient use of resources.

In Africa, the adoption of precision agriculture is growing, albeit at a slower pace compared to more developed regions [20]. The continent faces unique challenges, such as limited access to technology, high costs, and inadequate infrastructure [21]. However, there are promising developments. Countries like South Africa and Kenya are leading the way, utilizing technologies like mobile apps for weather forecasting, soil sensors, and satellite imagery to improve agricultural productivity [22]. Initiatives and partnerships with international organizations are crucial in promoting PA technologies across the continent. These efforts are helping to address food security issues, improve crop management, and empower smallholder farmers through better access to data and resources.

With its largely agrarian economy, Ethiopia is beginning to embrace PATs to enhance its agricultural sector [23]. In collaboration with various international agencies and NGOs, the Ethiopian government is working to introduce and scale up PA practices [24]. Efforts are focused on using satellite data for better land management, deploying soil sensors to monitor soil health, and employing drones for crop surveillance [25]. Despite these advancements, the widespread adoption of PA in Ethiopia is hindered by factors such as high initial costs, lack of technical expertise, and limited infrastructure [26]. Nevertheless, pilot projects and localized initiatives are showing positive results, indicating the potential for broader implementation in the future.

## 4. Applications of Precision Agriculture for Sustainable Crop Production and Environmental Conservation

### 4.1. Soil Management

Precision agriculture, leveraging advanced technologies such as GPS, remote sensing, and IoT devices, revolutionizes soil management by enabling farmers to implement site-specific interventions [27, 28]. This approach allows for detailed mapping of soil properties, such as pH, nutrient levels, and moisture content, across different areas of a field. Farmers can then tailor their soil management practices to address the unique needs of each section, optimizing inputs such as fertilizers and water [29]. This targeted approach increases crop yields and quality while improving resource efficiency and reducing environmental impacts such as nutrient runoff and groundwater contamination [30]. Moreover, precision agriculture facilitates continuous monitoring and data collection, providing real-time insights into soil health and enabling proactive management strategies [31]. Sensors and drones can detect early signs of soil degradation, pest infestations, or nutrient deficiencies, allowing for timely corrective actions [32]. Data analytics and machine learning predict soil behavior, helping farmers address issues before they escalate [33]. As a result, precision agriculture not only sustains soil productivity but also supports long-term soil health, contributing to sustainable farming practices and food security.

### 4.2. Water Management

Precision agriculture, a farming management concept that uses technology to monitor and optimize agricultural processes, has significantly advanced water management in farming [34]. This approach leverages data from various sources, such as soil moisture sensors, weather forecasts, satellite imagery, and IoT devices, to make informed decisions about irrigation practices [35]. By accurately assessing the moisture needs of specific areas within a field, farmers can apply water more efficiently, reducing waste and ensuring crops receive the optimal amount of hydration [36]. This not only conserves water but also enhances crop yields and reduces the risk of water-related diseases [37].

Moreover, precision agriculture's application in water management extends to the use of advanced irrigation systems such as drip irrigation and variable rate irrigation (VRI) [38]. Data-driven systems provide precise water delivery, minimizing evaporation and runoff, while VRI adjusts rates for different field zones [39]. This level of precision helps manage scarce water resources more effectively, promote sustainable farming practices, and mitigate the environmental impact of agriculture [40]. Generally, precision agriculture revolutionizes water management, making it more sustainable and efficient [17].

### 4.3. Crop Monitoring

Precision agriculture has revolutionized crop monitoring using advanced technologies such as GPS, remote sensing, and IoT devices [41]. With remarkable accuracy, these technologies enable farmers to collect detailed information about their fields, including soil health, moisture levels, and crop conditions [35]. Using satellite imagery and drones equipped with multispectral cameras, farmers can monitor crop health in real time, identifying areas affected by pests, diseases, or nutrient deficiencies [42]. These granular data allow for targeted interventions, such as variable rate application of fertilizers and pesticides, optimizing inputs, and minimizing environmental impact [43]. Consequently, precision agriculture enhances productivity and promotes sustainable farming practices by reducing the overuse of chemicals and conserving resources [44].

Furthermore, precision agriculture systems often integrate data analytics and machine learning algorithms to predict crop performance and identify trends [45]. By analyzing historical data alongside real-time observations, these systems can provide farmers with actionable insights to improve yield predictions and optimize planting schedules [46]. For instance, predictive models can forecast weather patterns and suggest optimal planting times to avoid adverse conditions [47]. This proactive approach reduces the risk of crop failure and increases overall farm efficiency [48]. In addition, mobile applications and cloud-based platforms facilitate seamless data sharing and remote monitoring, enabling farmers to make informed decisions from anywhere [49]. Ultimately, precision agriculture improves decision-making, boosts productivity, and enhances the sustainability and profitability of modern farming.

### 4.4. Nutrient Management

**Figure 2:**
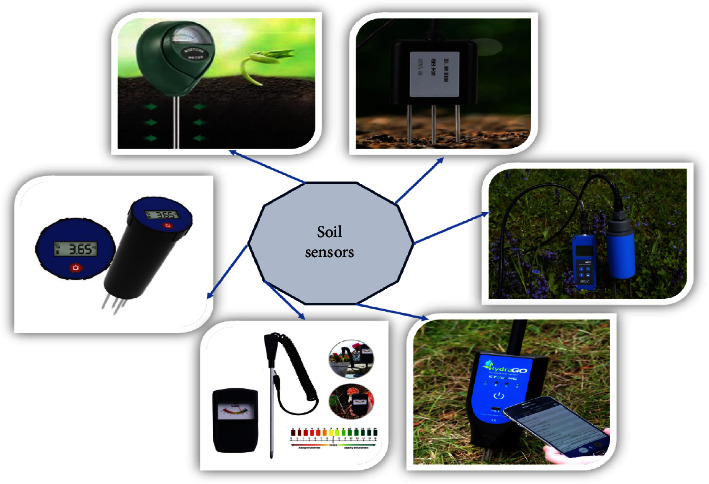
Different agricultural sensors used for soil nutrient management.

Precision agriculture, leveraging advanced technologies such as GPS, remote sensing, and data analytics, revolutionizes nutrient management in farming [50]. This approach enables the precise application of fertilizers and other soil amendments based on detailed, site-specific information about soil nutrient levels and crop needs [51]. Farmers can create variable rate application maps by integrating data from soil tests, crop yield monitors, and aerial imagery [52]. Remote sensing provides a nonintrusive and economical method for obtaining vital information about crop health and the nutrients contained in the soil or plants [53] (Figure 2). Using remote sensing to detect nutrient stresses and integrating this data into a Geographic Information System (GIS) can aid site-specific fertilizers and soil amendment applications in India [54]. This, in turn, would increase fertilizer use efficiency and reduce nutrient losses. Precision agriculture combines data from soil tests, remote sensing, and historical yield records to create nutrient management plans tailored to specific field zones [55]. This approach ensures that fertilizers are applied at optimal rates and timings, maximizing crop nutrient uptake while minimizing nutrient runoff and leaching [56].

Moreover, precision agriculture enhances environmental sustainability by mitigating the negative impacts of conventional farming practices [57]. Applying nutrients only where needed minimizes nutrient runoff into waterways, which can cause algal blooms and other ecological problems [58] (Table 1). In addition, it reduces greenhouse gas emissions associated with fertilizer production and application. Advanced monitoring and feedback systems enable ongoing enhancement of nutrient management strategies, allowing farmers to adjust their practices using real-time data and historical trends [71]. This adaptive approach ensures nutrient management is both cost-effective and ecofriendly, supporting the sustainability of agricultural ecosystems.

### 4.5. Pest and Disease Management

Precision agriculture applies advanced technologies and data management techniques to enhance pest and disease management in farming [62]. By integrating tools such as GPS, remote sensing, and GISs, farmers can monitor and manage crop health with high precision [72]. These technologies allow for the early detection of pest infestations and disease outbreaks by providing detailed spatial and temporal data [73]. For instance, remote sensing can identify subtle changes in crop color and vigor, indicative of stress or infection, which might be imperceptible to the naked eye [74]. By using such data, farmers can implement targeted interventions, reducing the need for blanket pesticide applications and thereby promoting more sustainable farming practices.

**Figure 3:**
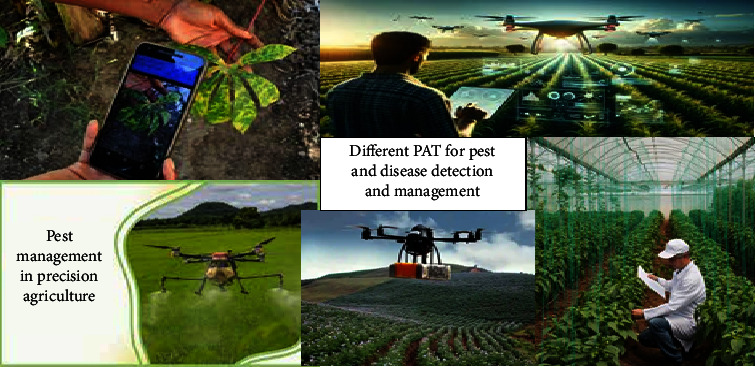
Different precision agriculture technologies for pest and disease detection and management.

One significant benefit of precision agriculture in pest and disease management is the ability to apply treatments more efficiently and effectively [64] (Figure 3). VRT enables the precise application of pesticides and fertilizers, tailored to the specific needs of different areas within a field [75]. This targeted approach optimizes resource use and minimizes environmental impact by reducing chemical runoff and soil contamination. Furthermore, precision agriculture can integrate predictive modeling, which uses historical data and current conditions to forecast pest and disease pressures [76]. This allows farmers to take proactive measures, such as adjusting planting schedules or selecting resistant crop varieties to mitigate potential issues before they become severe.

In addition to improving immediate pest and disease control, precision agriculture fosters long-term agricultural resilience [77] (Figure 3). By continuously collecting and analyzing data, farmers can gain deeper insights into the patterns and factors influencing pest and disease dynamics [78]. This knowledge supports the development of integrated pest management (IPM) strategies that emphasize ecological balance and sustainable practices [79]. For example, farmers can identify beneficial insect populations that naturally control pests and adjust their management practices to support these beneficial species [80]. In general, precision agriculture enhances the efficiency and effectiveness of pest and disease management and contributes to the sustainability and resilience of agricultural systems [64].

### 4.6. Planting and Harvesting

Precision agriculture has revolutionized both planting and harvesting processes, leveraging technology to optimize efficiency and yield while minimizing resource wastage [17]. In planting, precision agriculture uses advanced techniques like GPS-guided machinery and VRT to place seeds at optimal depths and spacing, ensuring uniformity across the field [81]. By analyzing soil data, including nutrient levels and moisture content, farmers can tailor their planting strategies to maximize the potential of each crop [82]. This targeted approach not only enhances yield but also reduces input costs by minimizing seed and fertilizer usage. During harvesting, precision agriculture continues to play a crucial role in streamlining operations and maximizing productivity [28]. Technologies such as yield monitoring systems and remote sensing allow farmers to monitor crop health and maturity in real time, enabling them to identify the optimal time for harvesting [83]. By integrating weather forecasts and soil data, farmers can optimize harvest timing and reduce losses from adverse weather or crop stress [76]. Furthermore, the use of advanced machinery equipped with sensors and automation technology enhances efficiency during harvesting, reducing labor requirements and increasing overall output [84].

Overall, precision agriculture applications in planting and harvesting revolutionize traditional farming practices, improving productivity, sustainability, and profitability [85]. By leveraging data-driven insights and advanced technologies, farmers can optimize every stage of the agricultural process, from planting to harvesting, resulting in higher yields, reduced environmental impact, and increased economic returns [86]. As the agriculture industry embraces innovation, precision agriculture is set to play an increasingly critical role in feeding a growing global population while preserving natural resources for future generations [62].

### 4.7. Climate Adaptation

Precision agriculture, with its ability to optimize resource use and maximize crop yields, is increasingly recognized as a vital tool in climate adaptation strategies for agriculture [87]. One key aspect of climate change is its impact on weather patterns, including shifts in precipitation, temperature, and extreme weather events [88]. PATs such as sensors, drones, and satellite imagery offer real-time monitoring and data collection capabilities, allowing farmers to understand better and respond to these changing conditions [89]. For instance, sensors can track soil moisture levels, enabling farmers to adjust irrigation schedules to match changing precipitation patterns, thus conserving water and mitigating drought risks.

Moreover, precision agriculture enables farmers to tailor inputs such as fertilizers and pesticides more precisely to the needs of specific areas within their fields [6]. This optimizes resource use, reduces environmental impact, and enhances resilience to climate change. By applying inputs only where and when needed, farmers can minimize waste and ensure that crops receive the nutrients and protection they require, even in the face of shifting environmental conditions [90]. In addition, the data collected through precision agriculture systems can be used to improve crop breeding programs, developing varieties that are better adapted to the challenges posed by climate change, such as heat and drought tolerance [91].

Furthermore, precision agriculture supports implementing climate-smart practices such as conservation tillage and cover cropping [92]. Accurate mapping of field characteristics allows farmers to create tailored conservation strategies, such as planting cover crops in erosion-prone areas or using no-till practices in regions with low soil organic matter. These practices mitigate climate change by sequestering carbon and enhance resilience by improving soil health and water retention, reducing vulnerability to extreme weather [93]. Overall, precision agriculture plays a crucial role in helping farmers adapt to the challenges of climate change while ensuring the sustainability and productivity of agricultural systems [57].

### 4.8. Data Management and Analysis

In precision agriculture, data management plays a pivotal role in streamlining the diverse array of information collected from various sources [94]. This includes soil sensors, satellite imagery, weather forecasts, and crop health-monitoring systems, integrated into cloud-based platforms for real-time farmer access and analysis [95]. These systems organize the data and ensure its accuracy and reliability, providing farmers with a solid foundation upon which to base their decisions.

Once the data are organized, analysis techniques such as machine learning and predictive analytics come into play [96]. Machine learning algorithms can sift through vast amounts of data to identify patterns and correlations that may not be immediately apparent to human observers [97]. For example, these algorithms can analyze historical weather data alongside crop yields to uncover relationships between specific weather patterns and agricultural productivity. On the other hand, predictive analytics use these identified patterns to forecast future outcomes, such as crop yields or pest outbreaks [98]. By leveraging these advanced analysis techniques, farmers can make data-driven decisions that optimize resource allocation, reduce risks, and ultimately improve overall farm performance.

### 4.9. Sustainable Land Management

Precision agriculture, with its focus on optimizing inputs and maximizing yields, has emerged as a powerful tool in sustainable land management [99]. By utilizing technologies such as GPS, sensors, drones, and machine learning algorithms, precision agriculture enables farmers to monitor and manage their fields with unprecedented accuracy [2]. This precision allows for the targeted application of resources such as water, fertilizers, and pesticides, reducing waste and minimizing environmental impact [3].

A key aspect of precision agriculture is supporting soil health and conservation by mapping field variability, enabling farmers to tailor practices to each area's specific needs [67]. This might involve adjusting irrigation schedules to match soil moisture levels, applying only fertilizers where and when needed, or implementing conservation tillage techniques to minimize soil disturbance [100]. These practices improve crop yields and help preserve soil structure, reduce erosion, and protect water quality.

Furthermore, precision agriculture facilitates adopting regenerative farming practices that promote long-term sustainability [101]. Continuous monitoring of soil health indicators such as organic matter, nutrient levels, and compaction helps farmers make informed decisions to improve soil fertility and resilience over time [102]. This approach benefits the environment by reducing emissions and conserving resources while boosting farm profitability and resilience to climate change [80]. In this way, precision agriculture plays a vital role in promoting sustainable land management practices that balance the needs of farmers, the environment, and future generations.

### 4.10. Carbon and Energy Management

Precision agriculture revolutionizes traditional farming practices by integrating cutting-edge technologies and data-driven strategies to optimize resource utilization and minimize environmental impact [28]. Central to its application is the precise monitoring and management of inputs such as water, fertilizers, and pesticides through advanced tools such as remote sensing, GPS-guided machinery, and sophisticated data analytics platforms [99]. This granular approach not only maximizes crop yields but also minimizes resource wastage, consequently reducing the carbon footprint associated with agriculture.

In addition to resource efficiency, precision agriculture facilitates the adoption of environmentally sustainable practices that promote carbon sequestration and energy conservation [3]. Techniques such as conservation tillage, cover cropping, and crop rotation, guided by soil and environmental data, improve soil health and boost carbon storage [103]. By reducing reliance on chemical inputs and optimizing land use, farmers can mitigate greenhouse gas emissions while simultaneously improving soil quality and resilience [104].

Furthermore, precision agriculture contributes to broader carbon and energy management efforts by providing valuable data for carbon accounting and emissions monitoring [105]. The detailed insights from precision agriculture systems enable farmers to accurately assess their carbon footprint and make informed decisions to mitigate emissions [106]. This data-driven approach supports regulatory compliance and allows farmers to participate in carbon markets and incentive programs that reward sustainable practices. Precision agriculture generally represents a paradigm shift in farming toward a more environmentally conscious and resource-efficient approach [107]. By optimizing every aspect of agriculture with technology and data, precision agriculture can drive sustainable development and address climate change and resource scarcity in food production [108]. Precision farming enhances crop production and environmental sustainability across various agricultural fields (Table 1).

## 5. Challenges of Precision Agriculture Application

Precision agriculture faces several global challenges while promising significant advancements in agricultural productivity and sustainability. First, the initial investment required for implementing PATs can be prohibitive for many farmers, particularly smallholders [18]. The cost of equipment such as GPS-guided machinery, sensors, drones, and the expenses associated with data collection and analysis pose significant barriers to adoption [109]. In addition, the lack of access to reliable internet connectivity and electricity in rural areas hampers the effective deployment and operation of precision agriculture systems [110].

In Africa, limited infrastructure and resources, such as inadequate road networks and storage facilities, intensify the challenges of transporting equipment and storing perishable produce [111]. Moreover, the variability of climate and soil conditions across the continent complicates the development of universally applicable precision agriculture solutions [112]. In some areas, political instability and conflict further impede technological advancements and investment in agricultural infrastructure [113].

Like many other African countries, Ethiopia grapples with similar challenges in adopting precision agriculture practices [114]. Despite its potential to revolutionize the country's agricultural sector, the high upfront costs and limited access to financing hinder widespread adoption [115]. Moreover, Ethiopia faces unique environmental and socioeconomic factors, such as land degradation and smallholder farming predominance, which require tailored precision agriculture solutions [116].

## 6. Review Gaps and Future Line of Works

The systematic review of PATs for sustainable crop production and environmental sustainability reveals several critical gaps. There is a pronounced disparity in the accessibility and adoption of these technologies between developed and developing regions. In many developing countries, particularly in Africa and parts of Asia, inadequate infrastructure, limited financial resources, and insufficient technical expertise hinder the widespread adoption of precision agriculture. Furthermore, the review indicates a lack of localized studies addressing the unique agricultural conditions and challenges farmers face in these regions. Most existing research is concentrated in developed countries, where conditions, resources, and infrastructure differ significantly from those in developing regions. To bridge these gaps, future research should focus on developing cost-effective and scalable precision agriculture solutions tailored to the needs of resource-limited settings. This involves designing affordable sensors, accessible data analytics platforms, and simple user interfaces that smallholder farmers can easily adopt and use. There is also a need for interdisciplinary research that brings together agronomists, engineers, data scientists, and social scientists to develop holistic solutions. Such collaborations can drive innovations that are not only technologically advanced but also socially and economically viable. In addition, integrating advanced data analytics, such as machine learning and artificial intelligence, can enhance the predictive capabilities of precision agriculture, providing real-time insights for crop management, pest control, and climate adaptation.

The gaps identified in the current research emphasize the need for targeted investments and policy interventions to accelerate the adoption of PATs. Governments, international organizations, and private sector stakeholders should collaborate to build the necessary infrastructure, provide financial support, and offer training programs to equip farmers with the skills to effectively utilize these technologies. The implications of advancing precision agriculture are far-reaching, with the potential to significantly increase crop yields, improve resource use efficiency, and reduce environmental impacts. By addressing these gaps and focusing on future research priorities, it is possible to improve resilient and sustainable agricultural system capable of meeting global food demand for future generations.

## 7. Conclusion and Recommendation

In conclusion, applying PATs for sustainable crop production and environmental sustainability highlights significant benefits. Precision agriculture enhances crop yield by optimizing input use through GPS-guided machinery, remote sensing, and VRT. These technologies allow site-specific management, ensuring that resources such as water, fertilizers, and pesticides are applied precisely where and when needed. This not only improves crop productivity but also minimizes waste and input costs. In addition, precision agriculture practices contribute to environmental sustainability by reducing the overuse of agrochemicals, thus mitigating their negative impacts on soil health, water quality, and biodiversity. However, adopting PATs comes with challenges, including high initial costs and the need for specialized knowledge and training, particularly among smallholder farmers in developing countries who face financial and infrastructural constraints. Governments and agricultural institutions should provide subsidies and support services to facilitate the adoption of PATs, especially for small-scale farmers. Collaboration between the public and private sectors can drive innovation and reduce costs, making PATs more accessible. By implementing these recommendations, the agricultural sector can achieve sustainable crop production and environmental conservation, contributing to global food security and ecological balance.

## Figures and Tables

**Table 1:** Application of precision farming across various fields (short summary).

**S/N**	**Precision farming application**	**Quantitative benefits**	**Local barriers addressed**	**References**
1	Precision farming enhances soil health and resource efficiency using advanced technology to promote sustainable crop production	Soil health improvements by 20%–30%; resource use efficiency up by 15%	Poor soil fertility; inefficient resource use	[44, 59]
2	Precision farming conserves water by employing sensor technology and data analysis to optimize irrigation practices for sustainable crop growth	Water usage reduction by 30%–50%; crop yield increase of 10–20%	Water scarcity; inefficient irrigation systems	[60, 61]
3	Precision farming employs drones, satellite imagery, and IoT sensors for real-time crop monitoring to enhance decision-making and maximize yields	Yield improvement by 10%–25%; reduction in input costs by 15%	Lack of real-time crop data; high input costs	[57, 62]
4	Precision farming optimizes nutrient usage through data-driven techniques for sustainable crop production	Nutrient use efficiency up by 20%; reduction in fertilizer costs by 25%	Overuse of fertilizers; high nutrient costs	[31, 63]
5	Precision farming employs data-driven strategies and remote sensing to manage pest and disease outbreaks effectively for crop protection	Pest/disease reduction by 20%–40%; crop loss reduction by 15–25%	Pest and disease management challenges	[64, 65]
6	Precision farming optimizes planting and harvesting through GPS guidance and data analysis for enhanced efficiency and higher yields	Yield increase by 15%–30%; fuel savings of 10–20%	Inefficient planting/harvesting; high fuel costs	[44, 66]
7	Precision farming utilizes climate data and predictive modeling to adapt crop management practices, ensuring resilience to climate change impacts for sustainable agriculture	Improved crop resilience by 20%; adaptation costs reduced by 10%	Climate change impacts; adaptation costs	[57, 67]
8	Precision farming maximizes crop production through advanced data management and analysis techniques	Crop production increase by 15%–25%; data management efficiency up by 20%	Low productivity; inefficient data management	[68]
9	Precision farming uses technology to optimize resource usage and minimize environmental impact, promoting sustainable land management	Reduction in land degradation by 20%; resource usage efficiency up by 15%	Land degradation; inefficient resource use	[44, 69]
10	Precision farming minimizes carbon emissions and optimizes energy usage for sustainable agriculture	Carbon emissions reduction by 15%–25%; energy use reduction by 10–20%	High carbon emissions; high energy costs	[57, 70]

## Data Availability

Data sharing is not applicable to this article as no new data were created or analyzed in this study.
